# Pleomorphic Dermal Sarcoma With Rare Metastasis to the Colon

**DOI:** 10.7759/cureus.114430

**Published:** 2026-08-12

**Authors:** Nida Ahmed, Georgeanne Cornell, Danielle H Carpenter, Gillian Heinecke

**Affiliations:** 1 Department of Dermatology, SSM Health - Saint Louis University Hospital, St. Louis, USA; 2 Department of Dermatology, University of Nebraska Medical Center, Omaha, USA; 3 Department of Pathology, SSM Health - Saint Louis University Hospital, St. Louis, USA

**Keywords:** atypical fibroxanthoma, colon, metastasis, pleomorphic dermal sarcoma, skin cancer

## Abstract

A 76-year-old male presented with a firm ulcerated plaque on his right forehead. Initial superficial biopsy at an outside practice found an atypical dermal spindle proliferation suggestive of atypical fibroxanthoma (AFX). The superficial nature of the specimen limited subcutaneous evaluation. The patient was referred for Mohs micrographic surgery, and the specimen underwent pathologic evaluation. This specimen again revealed an atypical dermal spindle proliferation diffusely positive for CD10 and negative for CD34, Desmin, p63, pancytokeratin, and SOX10, now with infiltration into the subcutaneous tissue. A diagnosis of pleomorphic dermal sarcoma (PDS) was favored. Morphologically, PDS resembles AFX and shares many clinicopathologic features. It can be distinguished from AFX histologically by the presence of subcutaneous invasion, tumor necrosis, lymphovascular invasion, and/or perineural invasion. Ten months later, the patient presented with chest pain. Computed tomography scans revealed multiple lung nodules and a colon mass. Biopsy of the colonic lesion and a lung nodule indicated atypical spindle cells morphologically similar to the patient’s known PDS, consistent with metastatic disease. Early recognition and treatment of PDS is critical due to its high metastatic potential. This report highlights a unique case of colonic involvement, a rare manifestation with limited documentation in current literature.

## Introduction

Pleomorphic dermal sarcoma (PDS) is a rare malignant dermal neoplasm that typically presents on sun-exposed skin of elderly patients and is more common in males [1]. Morphologically, PDS is similar to atypical fibroxanthoma (AFX) and shares many clinicopathologic features [1,2]. It can be distinguished from AFX histologically by the presence of one or more of the following features: subcutaneous invasion, tumor necrosis, lymphovascular invasion, and perineural invasion [1,2]. Accurate identification of PDS is vital, as it has a greater rate of metastasis [1,2]. When PDS does metastasize, it most commonly spreads to the lungs and lymph nodes [1]. Due to its aggressive clinical course, early recognition of PDS is imperative for improved patient outcomes. We present a case of PDS of the forehead with unusual metastasis to the colon.

## Case presentation

**Figure 1:**
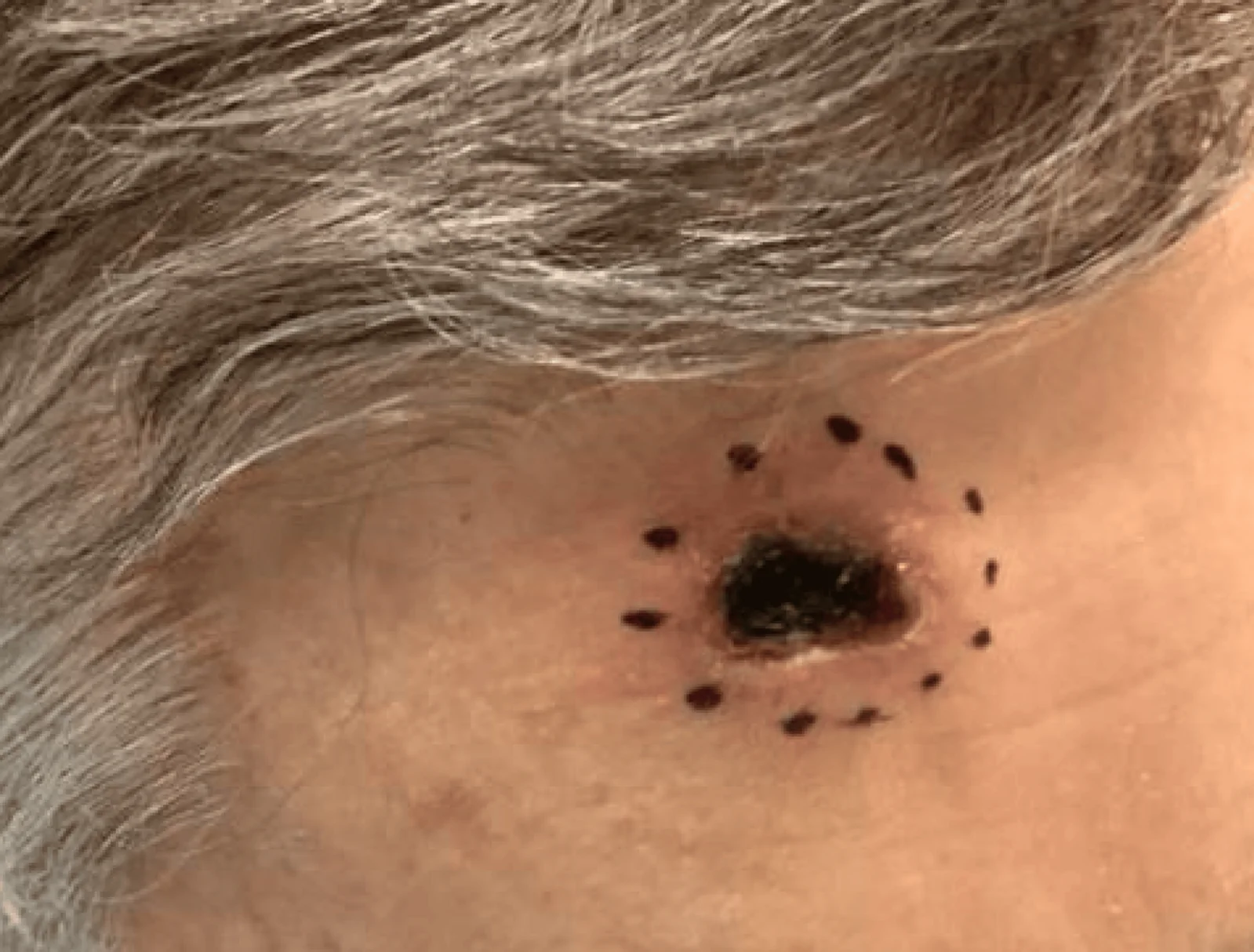
An ulcerated, pink plaque on the right superior forehead.

**Figure 2:**
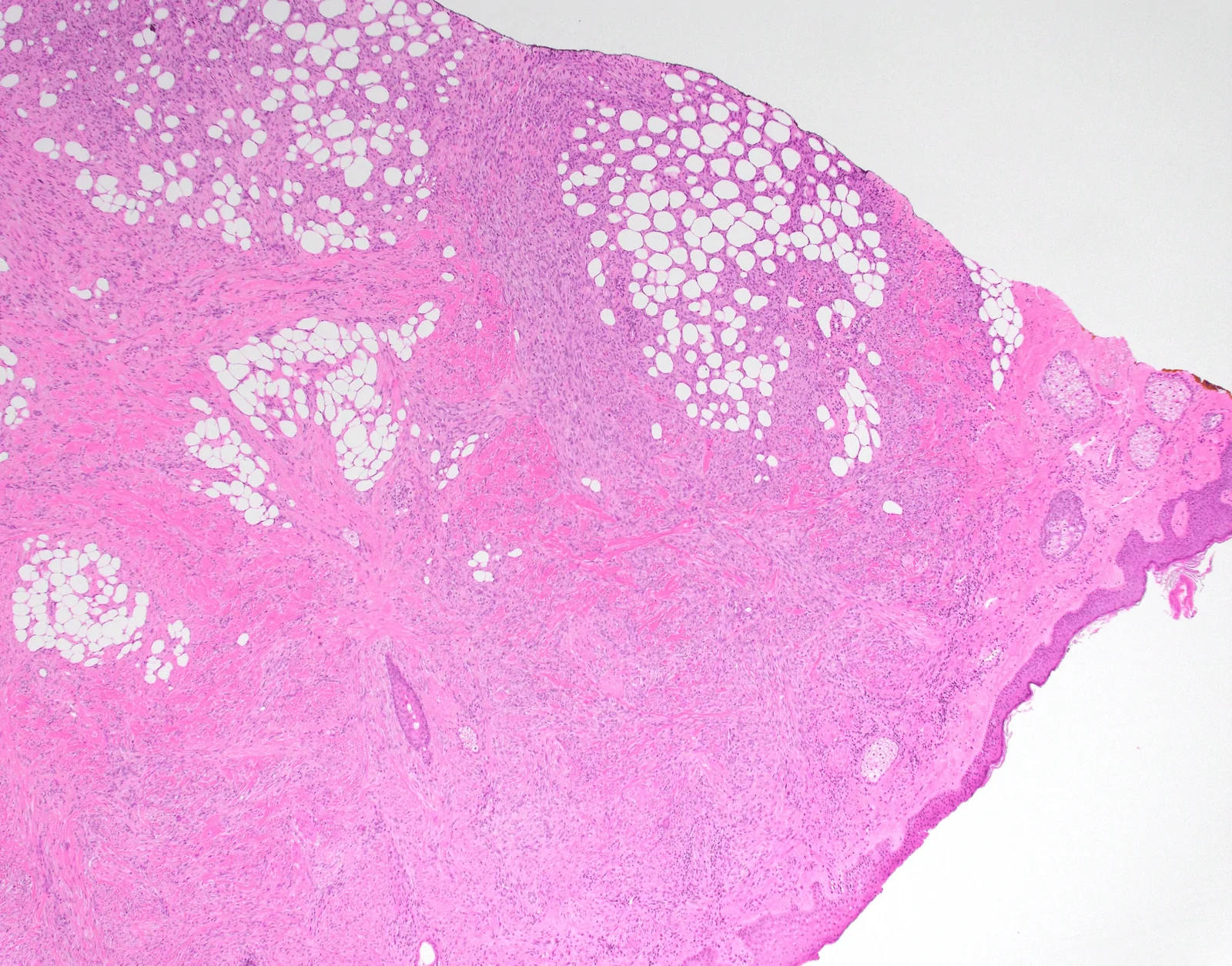
The debulk specimen demonstrating a dermal-based spindle cell tumor that infiltrates into the subcutaneous fat. (H+E, x40 magnification).

**Figure 3:**
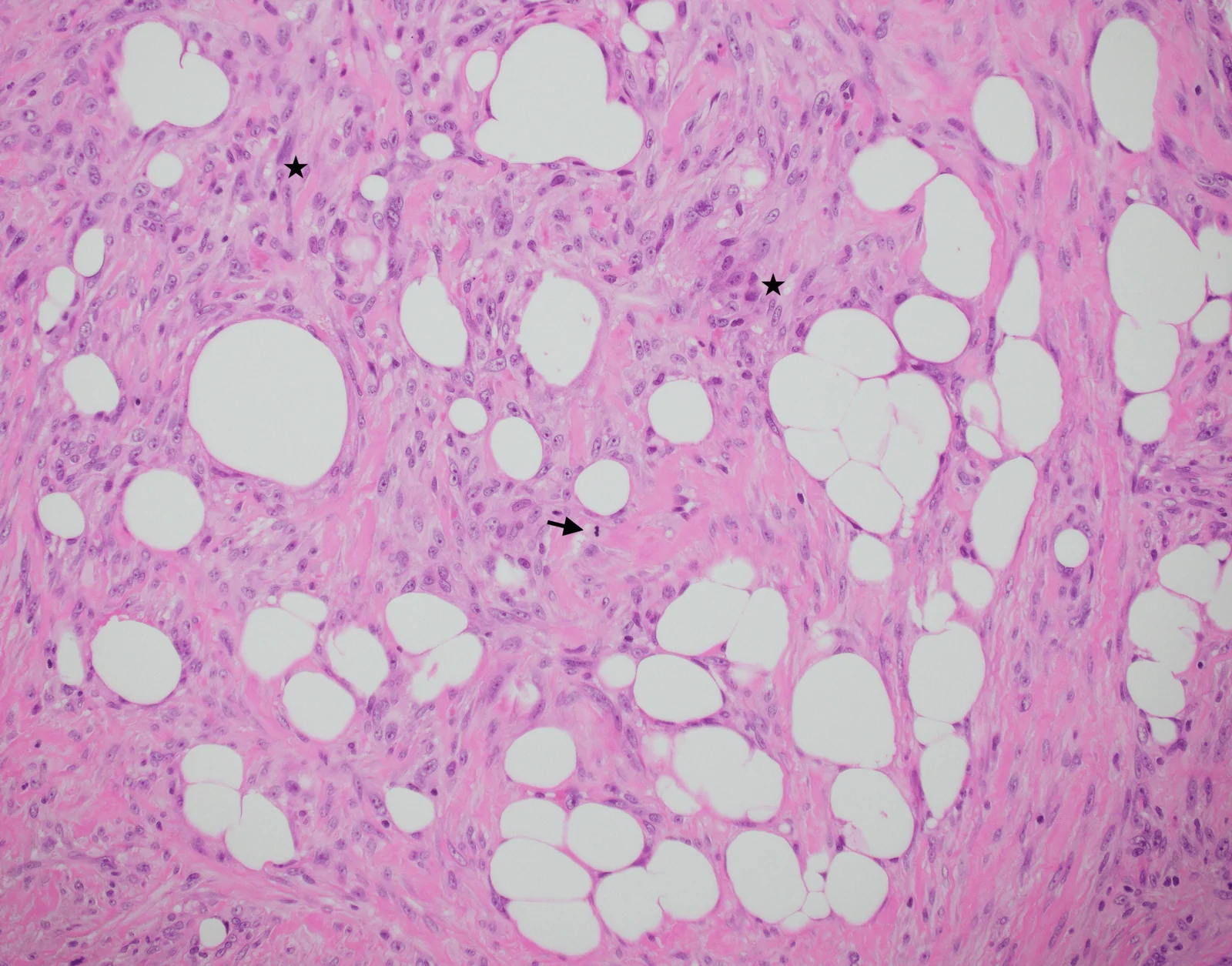
The spindle cell proliferation with nuclear hyperchromasia and pleomorphism (stars). Numerous mitotic figures are present (arrows) (H+E, x200 magnification).

**Figure 4:**
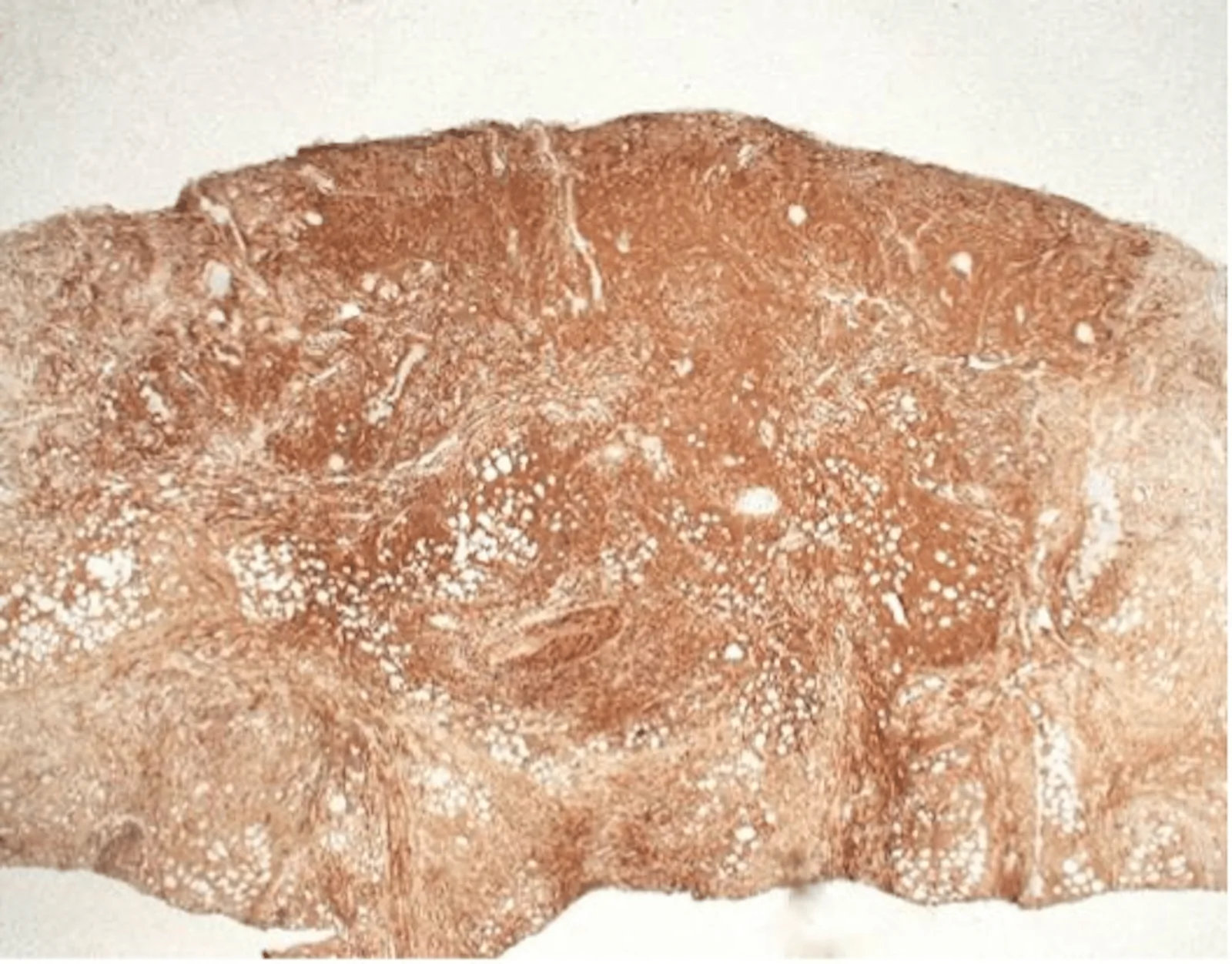
The tumor is diffusely positive for CD10 immunohistochemical stain (CD10 IHC, x100 magnification).

**Figure 5:**
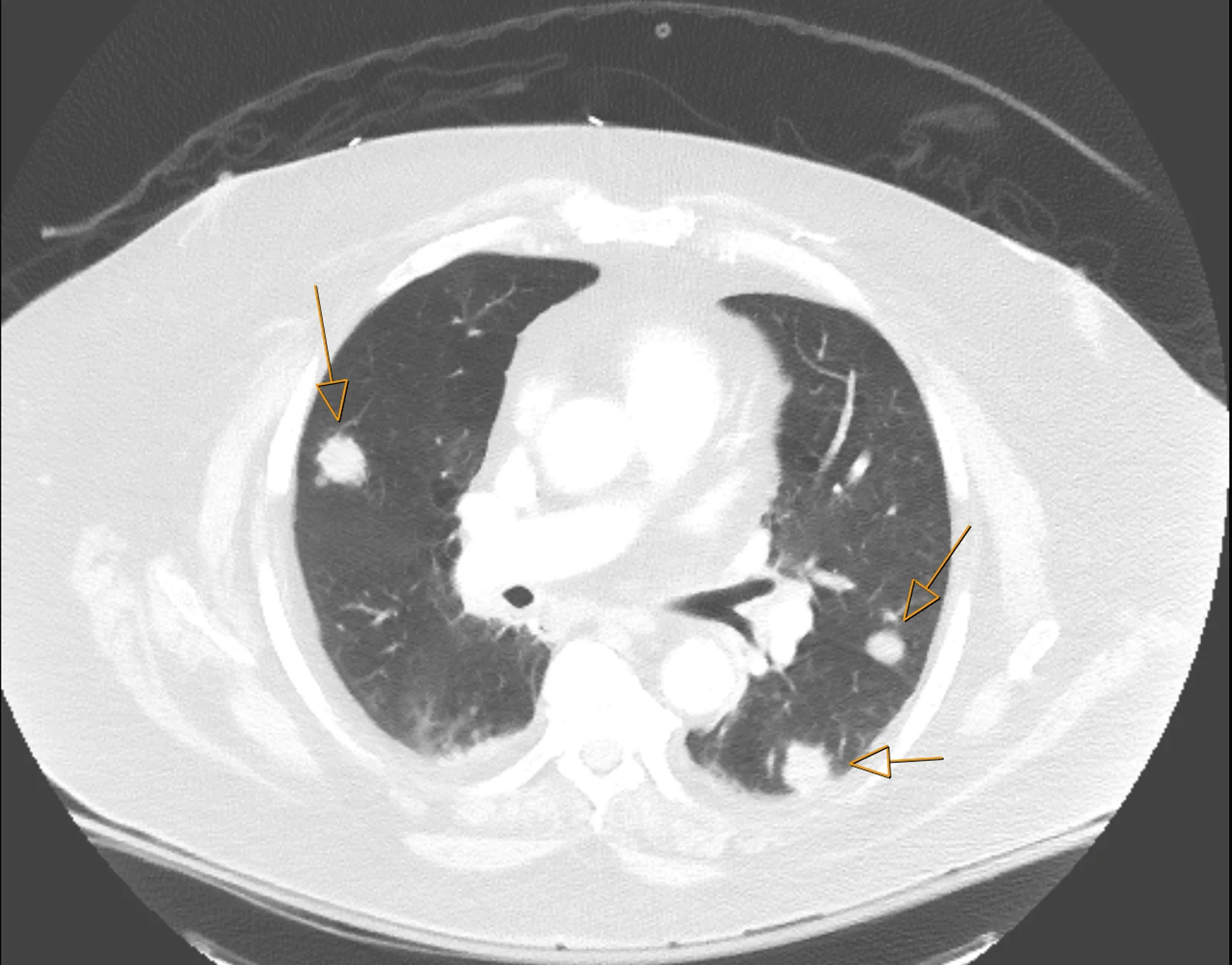
Computed tomography of the chest demonstrating multiple lung nodules (arrows).

**Figure 6:**
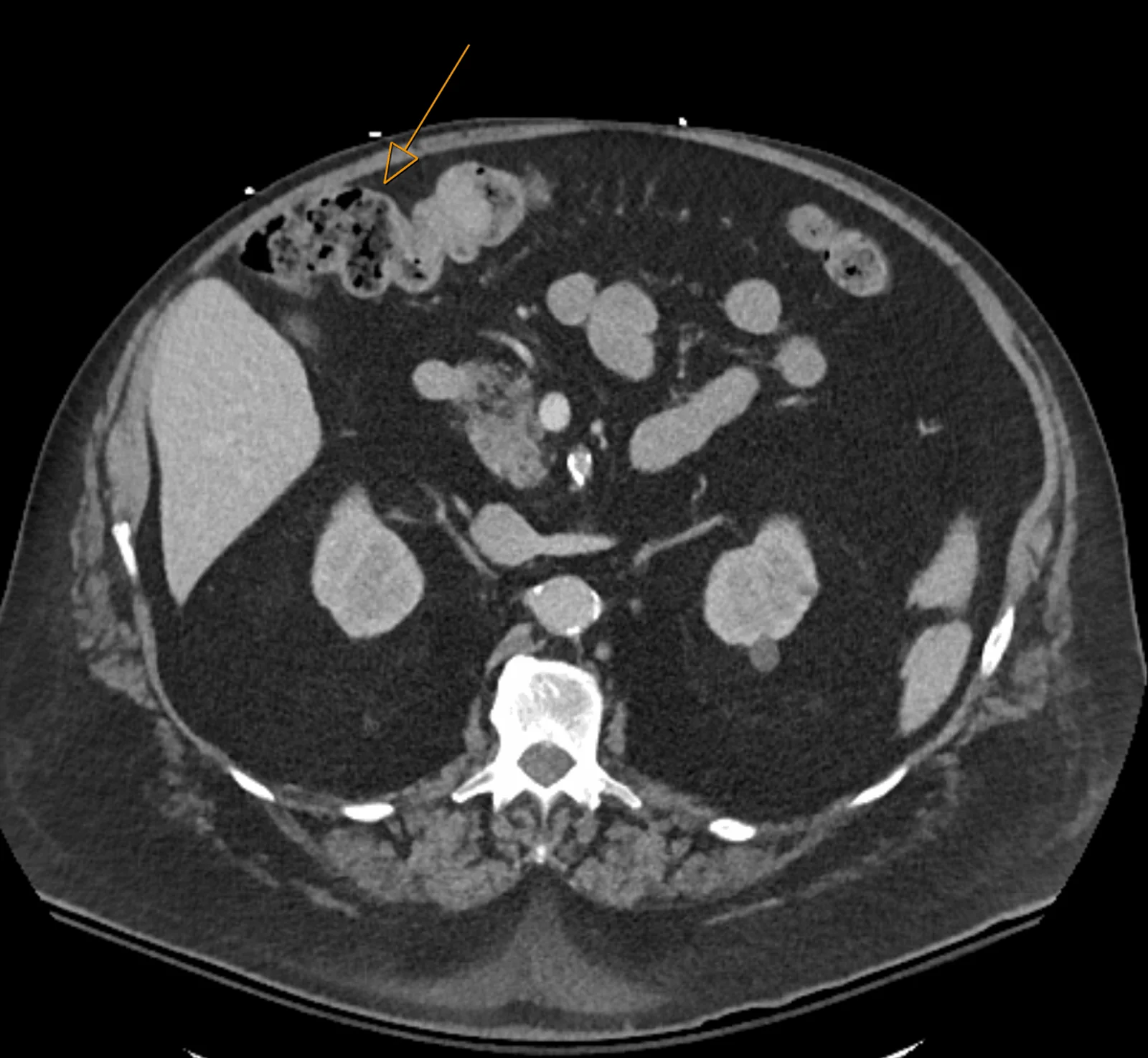
Computed tomography of the abdomen demonstrating a 2.9 cm by 2.9 cm transverse colon mass (arrow).

**Figure 7:**
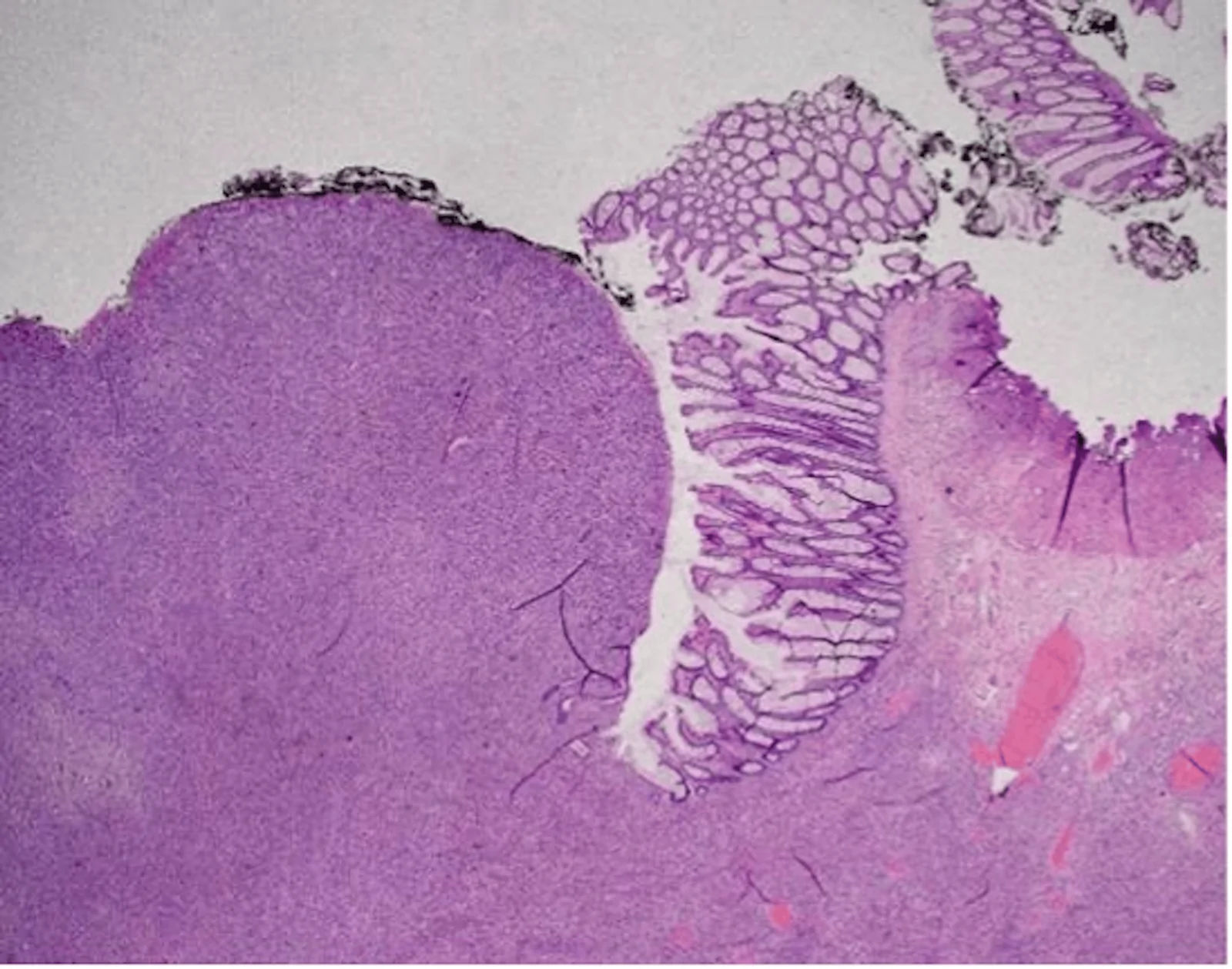
A spindle cell proliferation within the colon. (H+E, x100 magnification)

**Figure 8:**
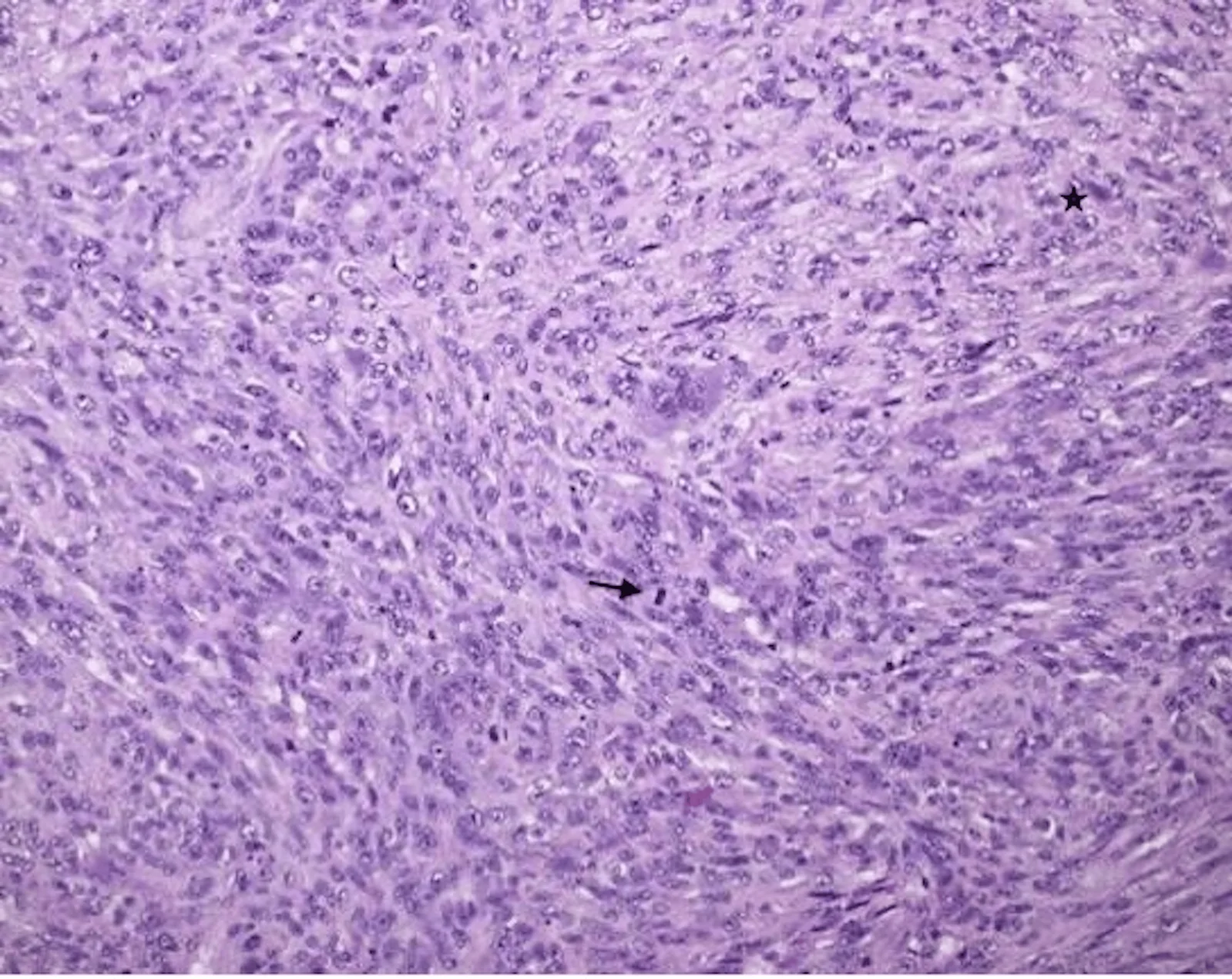
The spindle cell proliferation demonstrates pleomorphic cells (stars) with numerous mitotic figures (arrow). (H+E, x200 magnification)

A 76-year-old male presented with a firm, pink, ulcerated plaque on the right forehead (Figure 1). A shave biopsy at an outside facility revealed a dense dermal infiltrate of spindle cells with nuclear pleomorphism and hyperchromasia. Numerous mitotic figures were seen. The lesion was transected at the base of the specimen, limiting deep dermal and subcutaneous evaluation. The spindle cells were negative for MART-1, SOX10, pancytokeratin, high-molecular-weight cytokeratin, and CD34. There was weak positivity with factor XIIIa and CD68. Given the available histopathology and immunohistochemical findings, a diagnosis of AFX was rendered. The patient was referred to our institution for Mohs micrographic surgery, and the debulk specimen was sent for pathologic evaluation. The specimen revealed similar atypical spindle cell proliferation as reported in the prior biopsy; however, there was infiltration into the subcutaneous tissue (Figures 2, 3). The tumor was diffusely positive for CD10 (Figure 4) and negative for CD34, Desmin, p63, pancytokeratin, and SOX10. SMA and factor XIIIa demonstrated patchy positivity. Because of the infiltration into the subcutaneous fat, a diagnosis of PDS was favored. Ten months after his diagnosis, the patient presented to the emergency department with chest pain. Computed tomography of the chest, abdomen, and pelvis demonstrated multiple lung nodules and a 2.9 cm by 2.9 cm transverse colon mass (Figures 5, 6). A colonoscopy was performed for evaluation and revealed the aforementioned colon mass in addition to multiple colonic polyps. Pathologic evaluation of all colon lesions in addition to one of the lung nodules revealed a malignant spindle cell proliferation morphologically similar to the patient’s known PDS, consistent with widespread metastatic disease (Figures 7, 8). Due to the patient’s multiple comorbidities, he was ineligible for systemic therapy and passed away within the following year. 

## Discussion

PDS and AFX exist on the same biological spectrum, although it is imperative to correctly differentiate these diagnoses as PDS has a more aggressive clinical course. PDS is a large tumor with an infiltrative growth pattern and invasion into the subcutaneous tissue, distinguishing it from AFX [1-5]. Other features that, when present, distinguish it from PDS include tumoral necrosis, perineural invasion, and lymphovascular invasion [1,2]. Histologically, PDS presents as an atypical, pleomorphic, spindled, and epithelioid cell infiltrate. Multinucleated giant cells and mitoses, including many atypical forms, are commonly present [2]. Due to its morphologic similarities with other carcinomas and sarcomas, PDS is a diagnosis of exclusion, requiring a broad immunohistochemical panel to differentiate it from melanoma, sarcomatoid squamous cell carcinoma, leiomyosarcoma, and angiosarcoma [1,2,4,5]. Additionally, PDS and AFX commonly demonstrate CD10 positivity; however, CD10 is a nonspecific immunohistochemical marker that is not considered diagnostic of the tumor [2,3,5].

Surgical excision is the primary treatment approach for PDS, with Mohs micrographic surgery considered in select cases [2,6]. By contrast, Mohs surgery is commonly used as first-line treatment for AFX [6]. In the setting of unresectable or metastatic disease, chemotherapy and/or radiation may be indicated [2,6]. Emerging literature suggests a potential role for imaging and sentinel lymph node biopsy in the setting of PDS, as it exhibits relatively deeper infiltration with higher risk of metastasis [6]. After diagnosis of PDS, it is imperative to perform routine skin examinations and review of systems every three to six months to monitor signs of local recurrence or metastatic disease [2,6].

AFX has a metastatic potential of 1-2% while PDS displays a metastatic potential of 10-20% [1-5]. Metastasis of PDS most commonly occurs in the lungs or lymph nodes [1]. Metastasis to the colon is not reported in the literature. This case highlights a rare site of metastatic spread, stressing the importance of close monitoring in patients with a history of PDS.

There is limited information in the literature about the prognosis of PDS. Franzetti et al. retrospectively reviewed patient outcomes with PDS and AFX between 2012 and 2022 and found an increased mortality rate of patients with PDS in comparison to those with AFX, with rates of 60% and 26.5%, respectively [4]. Our case highlights that although these are cutaneous lesions and commonly treated by dermatologists, they may behave aggressively, and a multidisciplinary approach may be required in some instances.

## Conclusions

This case highlights the widespread metastatic potential of PDS with its rare involvement of the colon. The distinction between PDS, AFX, and other dermal spindle cell neoplasms is critical, as misdiagnosis could underestimate the disease severity and prognosis. Given the increased potential of PDS metastasis and poor prognosis, early recognition, accurate histopathologic differentiation, and close surveillance are essential for optimizing patient outcomes. This report also underscores the need for further studies in larger cohorts to better understand the clinical course, risk factors, and treatment strategies for patients with this rare and potentially aggressive malignancy.
